# Magnetic Cell-Mimetic Droplet Microrobots with Division and Exocytosis Capabilities

**DOI:** 10.34133/research.0730

**Published:** 2025-06-03

**Authors:** Shimin Yu, Weiwei Zhang, Yongzhi Feng, Xiang Zhang, Chuanhua Li, Shengjun Shi, Haocheng Wang, Tianlong Li

**Affiliations:** ^1^ College of Engineering, Ocean University of China, Qingdao 266100, China.; ^2^ School of Mechanical and Power Engineering, Zhengzhou University, Zhengzhou 450001, China.; ^3^ State Key Laboratory of Robotics and System, Harbin Institute of Technology, Harbin 150001, China.; ^4^ National Center for International Joint Research of Micro-Nano Molding Technology, School of Mechanics and Safety Engineering, Zhengzhou University, Zhengzhou 450001, China.

## Abstract

The first challenge in building a living robotic system inspired by life evolution is how to replicate the original form of life—the cell. However, current microrobots mimic cell motion control but fail to replicate the functional biological activities of cellular systems. Here, we propose a strategy that programs microparticle swarms encapsulated in droplets at an air/liquid interface to create cell-mimetic droplet microrobots with vitality by employing alternating magnetic fields. Through the design of algorithms and spontaneous interface waves, our collective system embodies reversible transitions between gas, chain, array, and disk-like collective modes, and emulates various complex activities of living cells in nature, including division and exocytosis. Based on these 2 capabilities learned from living cells, the cell-mimetic microrobots navigate the bile duct to the gallbladder under the guidance and control of magnetic fields, completing the drug release task. This cell-mimetic microrobots may provide a fundamental understanding of cellular life and pave the way for the construction of artificial living systems. Furthermore, they hold substantial potential for medical and environmental applications.

## Introduction

Nature has evolved from unicellular organisms to complex life through the struggle for survival, endowing cells with diverse functions [1]. For example, cell division enables organisms to grow by increasing their cell numbers and facilitating tissue development [2]. Damaged tissues or organs generate new cells through cell division for repair and regeneration [3]. To communicate and interact with their surroundings, cells secrete signaling molecules [4], hormones [5], and enzymes [6] via exocytosis, thereby regulating metabolism and maintaining physiological homeostasis. Scientists have long been intrigued by constructing artificial robotic systems with vitality [7–14]. However, the primary challenge for roboticists in replicating life along the path of natural evolution remains how to fully replicate the original form of life—the cell.

Droplets with phase separation and dynamic deformation properties similar to biological cells are often used to simulate and study fundamental processes in living cells. Specifically, custom droplets and intelligent soft materials designed for reconfigurability and adaptability to external stimuli (such as light [15–17], thermal [18], chemical [19–21], acoustic [22–24], electrical [25], and magnetic fields [26,27]) serve as ideal platforms for cell-mimetic microrobots. Additionally, the physical and chemical properties of liquids can endow droplets with functional and interfacial activity necessary for living robotic systems [28,29]. Magnetic actuation is a promising method for enabling the functionality of droplet microrobots due to its advantages such as accessibility, noncontact activation, biocompatibility, and ease of programming [30–34]. Magnetic fields are widely used for manipulating droplet microrobots, including deformation [35], locomotion [36,37], assembly [38,39], and biomimetic processes such as exocytosis or division [40,41]. They are also utilized in generating and transforming droplet swarms [42,43]. Throughout the development of magnetic droplet microrobots, advancements have progressed from initial unique shape designs [44,45] to externally controllable deformation capabilities [46,47] and further to customized magnetic field manipulation for collective robot control [48–50], all aimed at enhancing their ability to perform in vivo tasks. This evolution has also led to the emergence of various scalable and customizable fabrication techniques, as well as navigation control strategies in both open and confined environments [51,52]. Nevertheless, existing droplet microrobots continue to function as predefined units in task execution, facing inherent limitations in surpassing the physiological constraints of living organisms, and few efforts have been made to build artificial robotic systems capable of replicating the physiological activities of living cells or multicellular organisms.

**Fig. 1.**
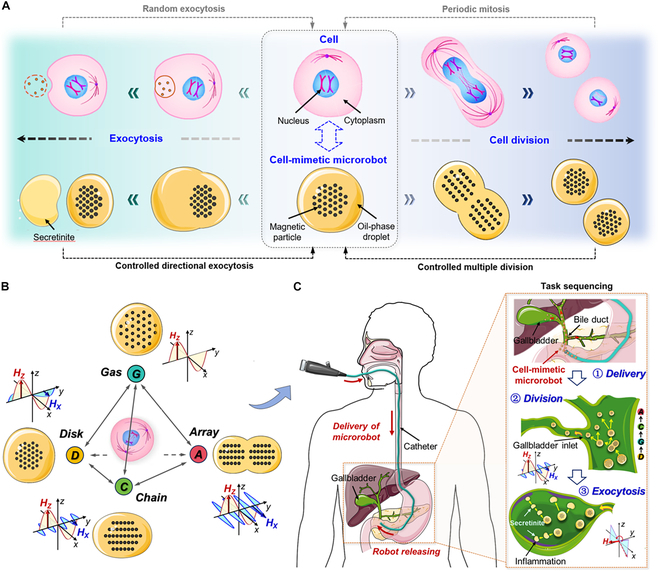
Multimode deformation behaviors and biomimetic activities of cell-mimetic microrobots. (A) Division and exocytosis behaviors of cell-mimetic microrobots imitating living cell. (B) Schematic of 4 programmable formations of cell-mimetic microrobots. Cell-mimetic microrobots were prepared by incorporating magnetic particles into oil droplets floating on the water surface. Four formations—gas (*G*)-like, chain (*C*)-like, array (*A*)-like, and disk (*D*)-like microparticle swarms—were programmatically triggered by combined orthogonal magnetic fields. (C) Schematic showing an example of the active catheter-based delivery process to the difficult-to-access bile duct. The inset shows the division and exocytosis activities of cell-mimetic microrobots that emulate natural cellular life.

Here, we create a cell-mimetic microrobot using pulsed magnetic fields to control magnetic droplets encapsulated at the air/liquid interface, which exhibit behaviors similar to those of living cells, such as division and exocytosis (Fig. 1A). As shown in Fig. 1B, various magnetic field strategies are employed to manipulate the reconfigurable magnetic particle swarm inside the cell-mimetic microrobot, forming reversible gas, chain, array, and disk-shaped patterns. This enables the microrobots to replicate unique life activities of natural cells. Furthermore, Fig. 1C illustrates the integration strategy for rapid and precise delivery of the cell-mimetic microrobots to the inaccessible gallbladder for targeted therapeutic interventions. The microrobots can be remotely and minimally invasively injected to a region near the bile duct through a catheter-equipped endoscope. Subsequent navigation of the microrobots is guided by magnetic actuation with real-time tracking via the endoscopic view. Experiments demonstrate the high adaptability and functionality of the cell-mimetic microrobots, including complex locomotion in narrow paths and channels through “division-like” behavior based on programmable formations, and targeted drug delivery to the gallbladder via “exocytosis-like” behavior. This controllable cell-mimetic microrobots offer a new design approach for the application of microrobots in deep tissues and organs with complex and variable cavities.

## Results

### Mechanism of multimode deformation behaviors

**Fig. 2.**
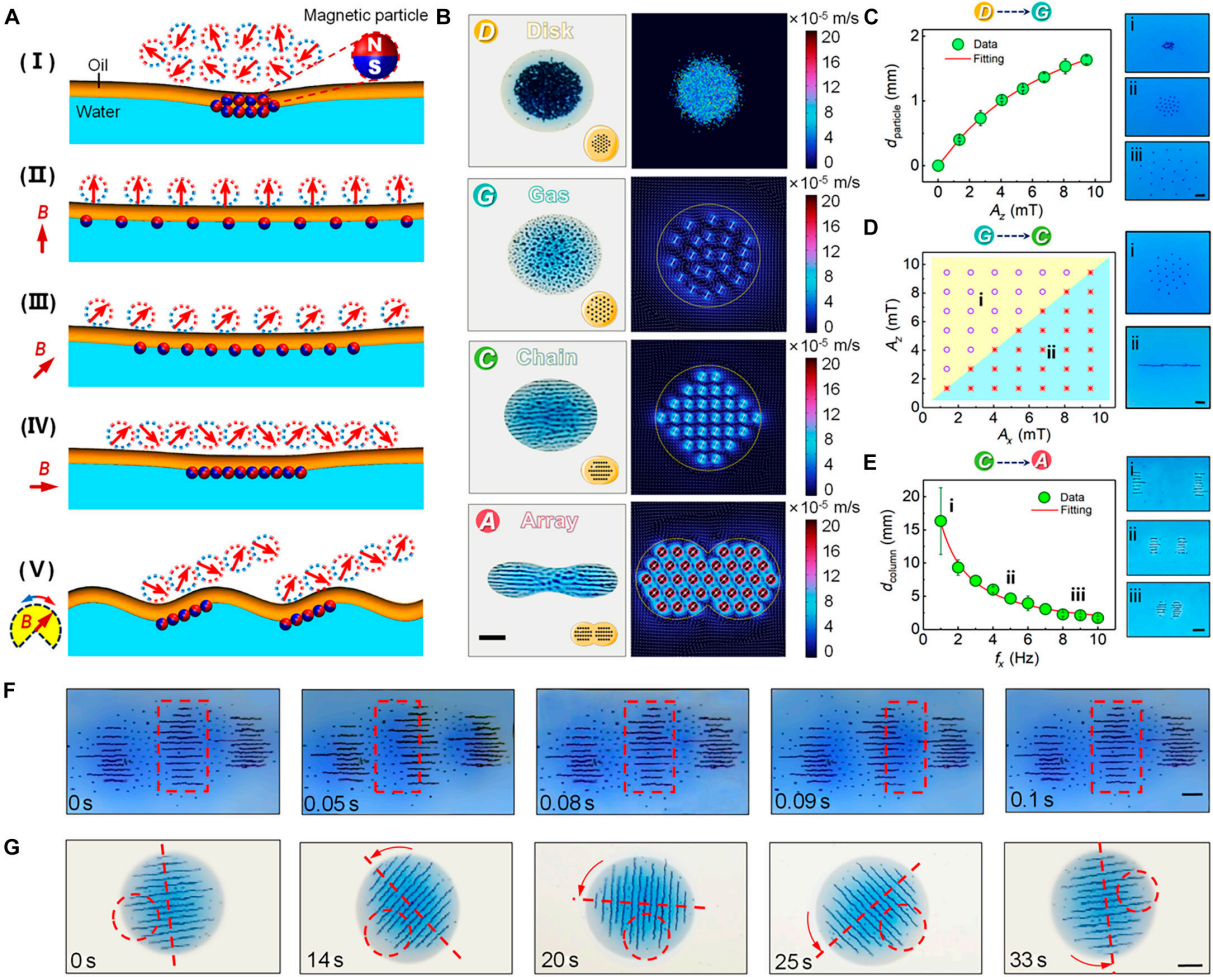
Actuation of magnetic cell-mimetic microrobots using combined orthogonal magnetic fields. (A) Schematic generation and division process of spindle fibers. The red and blue beads indicate the magnetization of the microparticles. To highlight the magnetization structure, arrows pointing in the same direction as particle magnetic moment are enlarged and placed above the particle clusters. (B) Snapshots from top to bottom showing patterns of the disk, gas, chain, and array in the cell-mimetic microrobots system. The simulation results of the flow field induced by an array of oscillating spheroids driven by different magnetic field. Scale bar, 4 mm. (C) Average distance of microparticles *d*_particle_ versus the amplitude *A*_z_ of the constant vertical field in (A-II). Scale bar, 1 mm. (D) The phase diagram presents 2 collective patterns actuated by different tilted field in (A-III). Scale bar, 0.5 mm. (E) Average distance of chain columns *d*_column_ versus the driving frequency *f*_x_ of orthogonal oscillation field in (A-V). Scale bar, 1 mm. (F) Resonance process between liquid surface and chain array (*f*_x_ = 10 Hz). The dark areas indicate the troughs filled with oil dyed dark blue, and the light areas represent the crests of interfacial wave. Scale bar, 3 mm. (G) Demonstration of chains rotating without moving against each other. Scale bar, 2 mm.

In order to further clarify the mechanism of microrobotic mitosis, a series of experiments were performed on the formation and division of “spindle fiber”. The division of cell-mimetic microrobots is triggered by the simultaneous application of 2 independent magnetic fields in the vertical and horizontal directions. To clearly represent this, we define the magnetic field strength and frequency in the vertical direction as ***A***_z_ and ***f***_z_, respectively. Similarly, ***A***_x_ and ***f***_x_ represent the strength and frequency of the magnetic field in the horizontal direction. Figure 2A schematically illustrates the generation process of reconfigurable magnetic particle swarm at the oil/water interface. In stage (i), microparticles aggregate at the oil–water interface because no magnetic field is applied in the present moment. At the beginning of stage (ii), the vertical field is actuated perpendicular to the liquid surface. Magnetic particles are oriented along the direction of external magnetic field and repel each other under the interaction of magnetic dipoles, generating uniformly distributed gas-like swarms. Driven by magnetic dipole repulsion, the average distance of particles increases with the field strength ***A***_x_ to balance the gravitational force of the particles on curved interface. After the superposition of a horizontal field in stage (iii), magnetic dipole attraction pulls the particles toward the center of the cluster. The tilted magnetic field does not alter the gas-like swarm pattern, until the amplitude ratio ***A***_x_/***A***_z_ reaches 1 [stage (iv)]. At this stage, as the magnetic particles aggregate into chain-like structures, they are simultaneously subjected to the magnetic torque exerted by both the external magnetic field and neighboring particles. Consequently, their magnetic axes no longer uniformly align with the external field direction but instead adopt a “zigzag” arrangement. This phenomenon is consistent with findings reported in previous studies [53]. Furthermore, we experimentally analyzed the variation of the distance between chains ***d***_chain_ with the magnitude of the magnetic field, and the results are shown in Fig. S1. The decrease of dchain with the increase of ***A***_z_ indicates that the vertical magnetic field produces a lateral attraction between the magnetic dipole chains (Fig. S1A). On the contrary, dchain increases with the increase of ***A***_x_, which indicates that the horizontal magnetic field can still induce and enhance the lateral repulsion of the magnetic dipole chain (Fig. S1B). This also proves that the magnetic dipoles of the particles in the chain are not arranged in a straight line, but in a “zigzag” arrangement with the head and tail connected. Then, the particle–particle attraction becomes stronger than the magnetic dipole repulsion, and adjacent particles aggregate into magnetic chains parallel to the applied field. Under the action of orthogonal oscillating field, the long chains first vibrate at the oil/water interface and then break down into shorter ones during stage (v), due to large angular velocity and the confinement of oil and water surface.

Fluidic interaction is another critical factor to trigger the formation of the magnetic chains. The fluidic velocity field induced by an oscillating particles or chain has been simulated. As shown in Fig. 2B, the magnetic particles gather toward the center and vibrate locally in “disk-like” mode with tiny magnetic field strength, similar to the Brownian motion, thus causing no noticeable flow of the surrounding liquid. When the magnetic field on the horizontal plane is small or disappears, the magnetic particles gather into short chains in the vertical direction and swing repeatedly under the excitation of the vertical oscillating magnetic field (OSMF) (“gas-like” mode). The induced flow always surrounds the swarm pattern, avoiding the swarm from contact with external objects, and therefore enhances its pattern stability as an entity. When the magnetic field on the horizontal plane is strong, the magnetic short chain oscillates repeatedly under the combined action of horizontal and vertical magnetic fields, with an increased oscillation angle. At this stage, the short chain aligns into a long chain along the direction of the horizontal magnetic field, causing the surrounding flow field to exhibit a noticeable ripple pattern (“chain-like” mode). By further increasing the frequency of the horizontal magnetic field, the oscillation frequency of the magnetic short chain also increases, leading to intense local changes in the flow field along the direction of the long chain. This, in turn, causes the long chain to fracture, gradually splitting the microrobot into 2 smaller microrobots (“array-like” mode). Flow field simulation analysis indicates that during the formation of magnetic particle swarm, magnetic interactions between particle chains are the main factor driving reconstruction. Additionally, fluid drag is also a cause of particle chain breakage under high-intensity, high-frequency magnetic field conditions.

To further elucidate the pattern of magnetic particle swarm changes for precise control of cell-mimetic microrobot modes, we analyzed the relationship between particle chain state transitions and magnetic field parameters. Figure 2C shows the process of switching from “disk-like” mode to “gas-like” mode of cell-mimetic microrobots in the experimental exploration. Driven by magnetic dipole repulsion, the average distance of particles ***d***_particle_ increases with the field strength ***A***_x_ to balance the gravitational force of the particles on the curved interface. After the superposition of a horizontal field in stage (iii), magnetic dipole attraction pulls the particles toward the center of the cluster. As shown in Fig. 2D-i, the tilted magnetic field does not alter the gas-like swarm pattern, until the amplitude ratio ***A***_x_/***A***_z_ reaches 1 [stage (iv)]. Then, the particle–particle attraction becomes stronger than the magnetic dipole repulsion, and adjacent particles aggregate into magnetic chains parallel to the applied field, which is also confirmed by the experimental results in Fig. 2D-ii. Under the action of orthogonal oscillating field, the long chains first vibrate at the oil/water interface and then break down into shorter ones during stage (v), due to large angular velocity and the confinement of oil and water surface. Figure 2E shows the inverse proportional relationship between the distance of chain columns ***d***_column_ and the driving frequency ***f***_x_, indicating that the oil–water interface fluctuates at the same frequency with magnetic chain array. Accordingly, it can be reasonably inferred that the resonance wave will drive magnetic chain array to split the surrounding oil droplet in turn, just like destroying the bridge resonant with the wind, which ultimately leads to the division of the cell-mimetic microrobot.

It is reasonable to assume that the magnetic dipoles of the particles in the chain are not arranged in a straight line, but a zigzag line connected head to tail [Fig. 2A(IV)]. In this way, the magnetic dipoles in 2 parallel chains are arranged in opposite directions (N-S-N to S-N-S). Therefore, transverse attraction of magnetic dipole chains would be generated by the opposite magnetic dipoles in chain pairs, and further strengthened by the vertical fields, resulting in magnetic field-induced contraction of particle chains as shown in Fig. 2F. Moreover, an additional experiment has been conducted to verify magnetization structure of the chains. During the rotation of parallel chains with the rotating magnetic field, no obvious mutual dislocation was observed even in the notch region presented in Fig. 2G. This can be reasonably explained by the lock-in effect of zigzag magnetic structure, the same as the coupling of magnetic gears.

### Division behavior of cell-mimetic microrobot

**Fig. 3.**
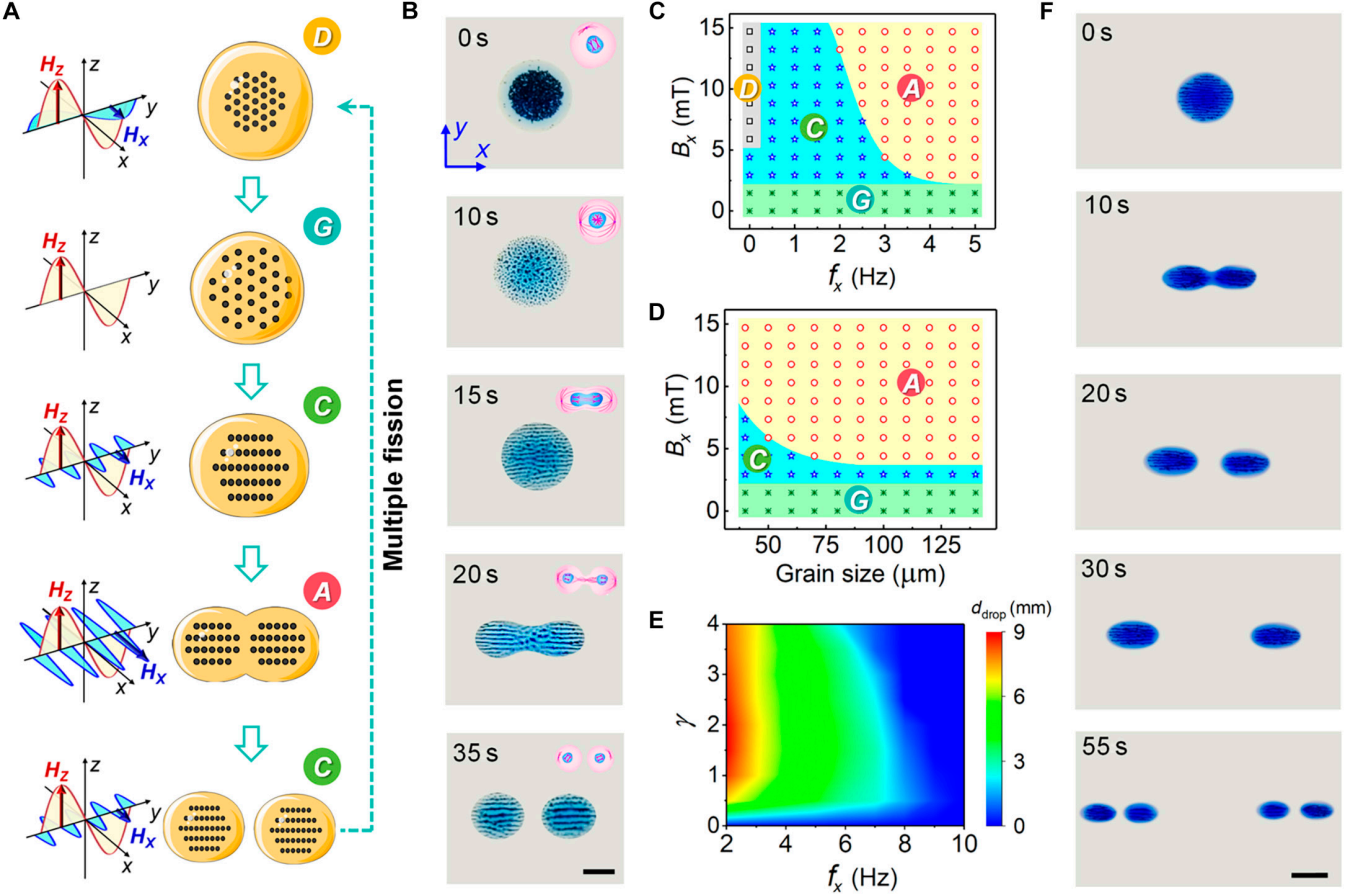
The division activity of cell-mimetic microrobots. (A) Schematic of division activity of cell-mimetic microrobots by switching formations. (B) Division process of cell-mimetic microrobot. Scale bar, 2 mm. (C) The phase diagram presents 4 collective patterns actuated by different OBMF. (D) The shape of the cell-mimetic microrobots pattern varies with the size of the magnetic particles inside. (E) Average distance of droplet microrobots versus the decreasing frequency *f*_x_ of the horizontal field with an amplitude of *A*_x_ = 10 mT. In (C) to (E), the microrobots are also subjected to a vertical alternating field (*A*_z_ = 10 mT, *f*_z_ = 0.5 Hz). All error bars indicate standard deviation (SD). (F) Demonstration of repeated robotic division. Scale bar, 4 mm.

Inspired by the cell division behavior of living cells, we developed a controllable division strategy for cell-mimetic microrobots (Fig. 3A). Meanwhile, the division process of a cell-mimetic microrobot is demonstrated in Fig. 3B and Movie S1. The magnetic field that triggers the microrobot’s division is an orthogonal butterfly magnetic field (OBMF), formed by the superposition of 2 distinct magnetic fields in the vertical and horizontal directions (as shown in Fig. S2A). By adjusting the parameters of these 2 fields, the internal magnetic particle modes and the splitting behavior of the robot can be effectively controlled. Initially, microparticles are concentrated in the center of oil droplet. When the vertical alternating field is applied, microparticles disperse uniformly to fill the entire droplet (*t* = 10 s). With the superposition of an orthogonal oscillating field of ***A***_x_ = 5 mT and ***f***_x_ = 1.5 Hz, adjacent particles aggregate into parallel chains that vibrate with the driving magnetic field at the oil–water interface (*t* = 15 s). As the oscillating field is strengthened (***A***_x_ = 10 mT, ***f***_x_ = 3 Hz), the column of chain-like swarm vibrates violently and finally splits in 2 chain-like swarms (*t* = 20 s). After a series of vibration processes, the division of cell-mimetic microrobot occurs like mitosis, forming 2 daughter microrobots (*t* = 35 s). The sub-droplets vibrate synchronously and repel each other on the water surface. Notably, the cell-mimetic microrobot’s unique “nucleus–cytoplasm” structure also grants it certain advantages over living cells. For example, after division, the cell-mimetic microrobots can be reassembled by adjusting the magnetic field direction in the horizontal plane (switching from ***A***_X_ to ***A***_Y_). Figure S3 illustrates this transformation process. This reversible division behavior provides a solution for the cell-mimetic microrobots to navigate through narrow tissue channels and exit the environment after completing its tasks.

The phase diagram in Fig. 3C presents the relationship between the pattern of cell-mimetic microrobots and the horizontal oscillating fields with different frequencies and amplitudes. In fact, it is the microparticles encapsulated in the microrobots that first respond to the external magnetic fields. Especially, magnetic particles will remain compactly aggregated if a constant magnetic field Bx is superimposed parallel to the water surface with a field strength of ***A***_x_ > 5 mT (region “*D*”). When ***A***_x_ is low, as shown in region “*G*”, the particles form gas-like patterns, which are loosely spaced and uniformly distributed in cell-mimetic microrobots. As ***A***_x_ increases (region “*C*”), chain-like swarms can be generated and spread over the entire microrobot. The chain swarms can be split into 2 column of chain array, followed by the division of cell-mimetic microrobots driven by the high-frequency fields in region “*A*”. That is, no division can be directly triggered from the original aggregated state by low-frequency fields. In addition, we also explored the effect of the size of magnetic particles on the pattern of cell-mimetic microrobots. From the experimental results in Fig. 3D, we can see that the pattern of the magnetic particle swarm inside the microrobots is less affected by the particle size, and the intensity of the horizontal magnetic field has a more obvious effect.

The analysis above indicates that, with the vertical magnetic field kept constant, the intensity and frequency of the horizontal magnetic field are the primary factors influencing the division of cell-mimetic microrobot. To facilitate controlled division of the microrobot, we define the average distance (*d*_droplet_) between the centers of the 2 resulting bodies as a measure to quantitatively describe how magnetic field intensity and frequency impact the division behavior. As shown in Fig. 3E, the distance between the 2 divided microrobots is related to both the strength (***A***_x_) and frequency (***f***_x_) of the horizontal magnetic field, which intuitively demonstrates that the horizontal magnetic field is the main factor triggering the splitting of the cell-mimetic microrobots. The magnetic strength ratio (***γ***) is defined as the ratio of the vertical magnetic field strength (***A***_z_) to the horizontal magnetic field strength (***A***_x_).

According to this principle, we further conduct experiments on multiple rounds of robotic division as shown in Fig. 3F and Movie S2. After the first round of division at *t* = 20 s, the separation of daughter microrobots is firstly expanded by a low frequency of 2 Hz to obtain sufficient reproductive space (*t* = 30 s). Then, with the activation of local resonance waves induced by oscillating fields of ***f***_x_ = 6 Hz, the second round of robotic division is realized as scheduled, regenerating 4 granddaughter microrobots (*t* = 55 s). We can expect that repeated division like living cells will continue as long as there is sufficient mass and energy supply.

### Exocytosis of cell-mimetic microrobots

**Fig. 4.**
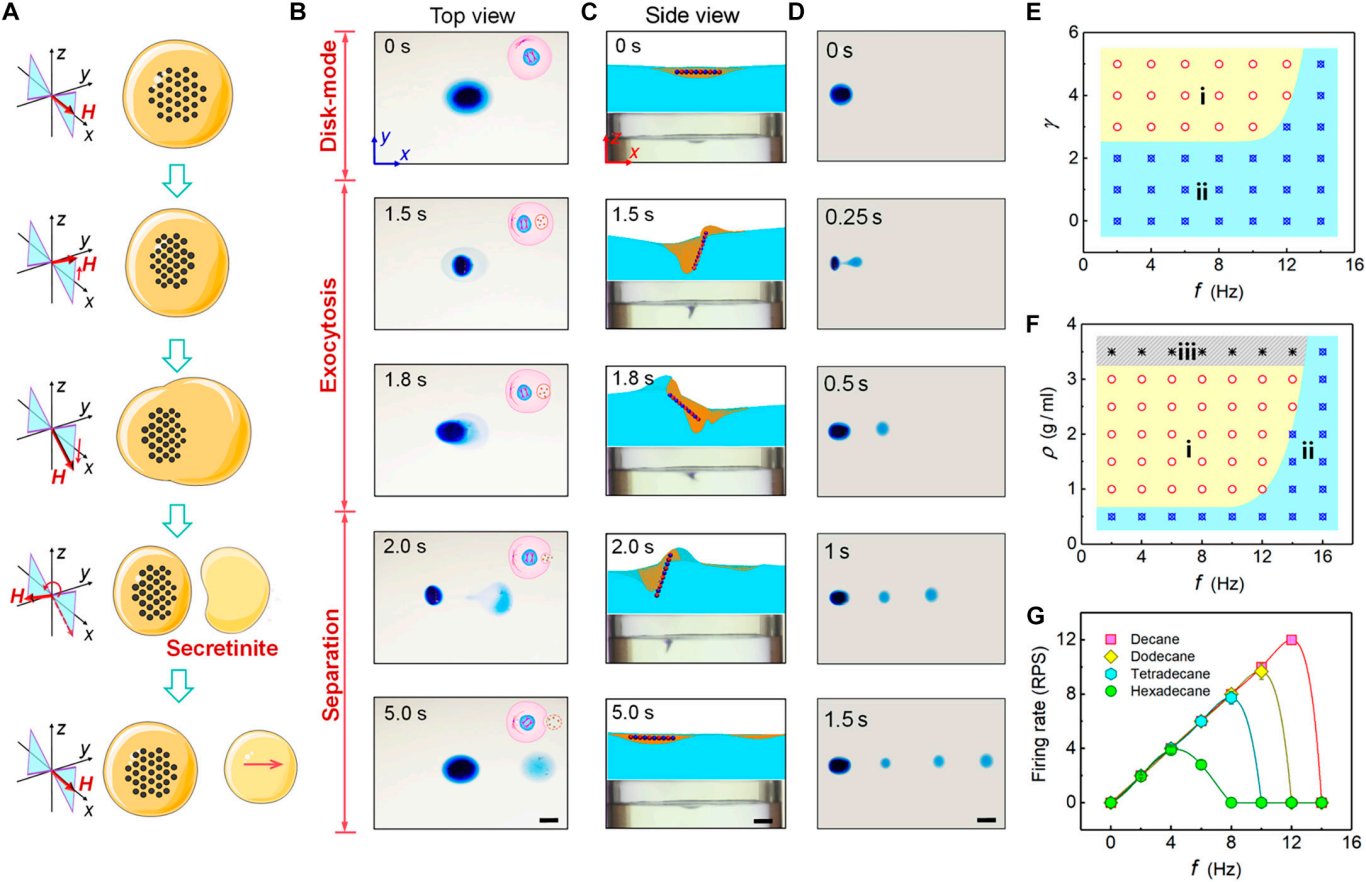
Robotic exocytosis activated by a combination of vertical magnetic fields and impulse surface waves. (A) Schematic exocytosis process of cell-mimetic microrobots floating on the water surface. (B) Exocytosis process of a cell-mimetic microrobot energized by butterfly oscillation fields of *γ* = 3 and *f*_x_ = *f*_z_ = 0.2 Hz. Scale bar, 2 mm. (C) Snapshots and schematic showing the mechanism of exocytosis process of a cell-mimetic microrobot floating on the water surface. Scale bar, 4 mm. (D) Repetitive multiple exocytosis process of a cell-mimetic microrobot excited by butterfly oscillation fields of *γ* = 3 and *f*_x_ = *f*_z_ = 2 Hz. Scale bar, 4 mm. (E) The phase diagram presents 2 exocytosis patterns actuated by oscillation fields of different inputs. Under magnetic fields in region (i), the microrobots cannot secrete daughter droplets. The exocytosis mode can form only in region (ii). (F) The phase diagram shows the relationship of exocytosis modes with field frequencies and microrobots’ densities. Microrobots with high density in region (iii) often fall from the water surface and sink to the bottom. (G) Firing rate of daughter droplets (returns per second) versus the increasing field frequency *f* for magnetic droplets of different alkanes.

Exocytosis, an important life activity of cells, can transport waste substances such as hormones and proteins to the exterior of the cell, or secrete chemical signal molecules to communicate with the surrounding cells. Here, the exocytosis behavior of cell-mimetic microrobots has also been activated through preprogrammed orthogonal OSMF, as shown in Fig. 4A. Figure S2B further illustrates the generation method of OSMF, which is also formed by the superposition of different magnetic fields in the vertical and horizontal directions. Under the activation of an incomplete butterfly oscillation fields, the cell-mimetic microrobots in “disk-like” mode sequentially undergoes eccentric displacement of magnetic particle clusters, deformation of the microrobot body, and partial separation, achieving a behavior analogous to cellular exocytosis. Figure 4B further illustrates the actual process of this exocytosis-like behavior exhibited by the cell-mimetic microrobots at a magnetic strength ratio (***γ*** = ***A***_z_/***A***_x_) of 3 and ***f***_x_ = ***f***_z_ = 0.2 Hz (Movie S3). To explore the mechanism behind the exocytosis-like behavior induced in the cell-mimetic microrobots under OSMF excitation, we recorded the exocytosis process from a frontal perspective (side view), considering that this behavior occurs on the water surface. As shown in Fig. 4C, schematic diagrams of the internal particle distribution at different time points have been added based on the recorded changes in the microrobot’s morphology for a more intuitive analysis of the internal mechanisms driving the exocytosis-like behavior. It can be observed that upon applying the magnetic field, the plane containing the magnetic particle cluster inside the microrobot tilts due to the magnetic moment, which induces deformation in the microrobot’s vertical plane and generates surface waves on the water. However, rapid magnetic field changes lead to continuous deformations of the microrobot. These new deformations cause direct collisions with the delayed surface waves generated by previous step, resulting in part of the microrobot’s body separating under shear forces and moving away with the surface wave. The experiments also revealed that the microrobot’s exocytosis-like behavior is not a one-time event but is repeatable. Furthermore, the frequency of excreted vesicles is directly correlated with the magnetic field frequency. As shown in Fig. 4D, when the frequency (***f***_x_) of OSMF was increased to 2 Hz, the microrobot achieved 3 consecutive exocytosis within 1.5 s.

To achieve precise control of the exocytosis behavior of cell-mimetic microrobot, we further explored the influence of 2 main parameters, OSMF parameters and internal particle density, on the exocytosis behavior. Figure 4E demonstrates the effect of the ratio of horizontal and vertical magnetic field strengths (***γ*** = ***A***_z_/***A***_x_) on exocytosis behavior under different magnetic field frequencies. The OSMF in the yellow region i cannot drive the microrobots to perform exocytosis, while the OSMF in the blue region ii can achieve this behavior. Figure 4F further reveals the effect of the density of magnetic particles inside the microrobots (***ρ***) on exocytosis behavior. It can be observed that the difficulty of achieving the exocytosis behavior increases with the density of magnetic particles, especially when the density exceeds 3 g/ml, as the microrobots are more likely to sink to the bottom of the water once the magnetic field is applied. Regarding the influence of magnetic field frequency, we quantitatively describe it through the microrobot’s exocytosis frequency. Figure 4G shows the exocytosis frequency of cell-mimetic microrobots composed of different matrices under various magnetic field frequencies. The exocytosis frequency increases with the rise in magnetic field frequency, and suddenly drops and eventually ceases altogether after reaching a certain point. This phenomenon resembles the “step-out” phenomenon observed in the microrobot’s motion speed as frequency increases. We refer to the magnetic field frequency at which the exocytosis frequency reaches its peak as the “break frequency”. For cell-mimetic microrobots composed of matrices hexadecane, tetradecane, dodecane, and decane, the break frequencies are 4, 8, 10, and 12 Hz, respectively.

**Fig. 5.**
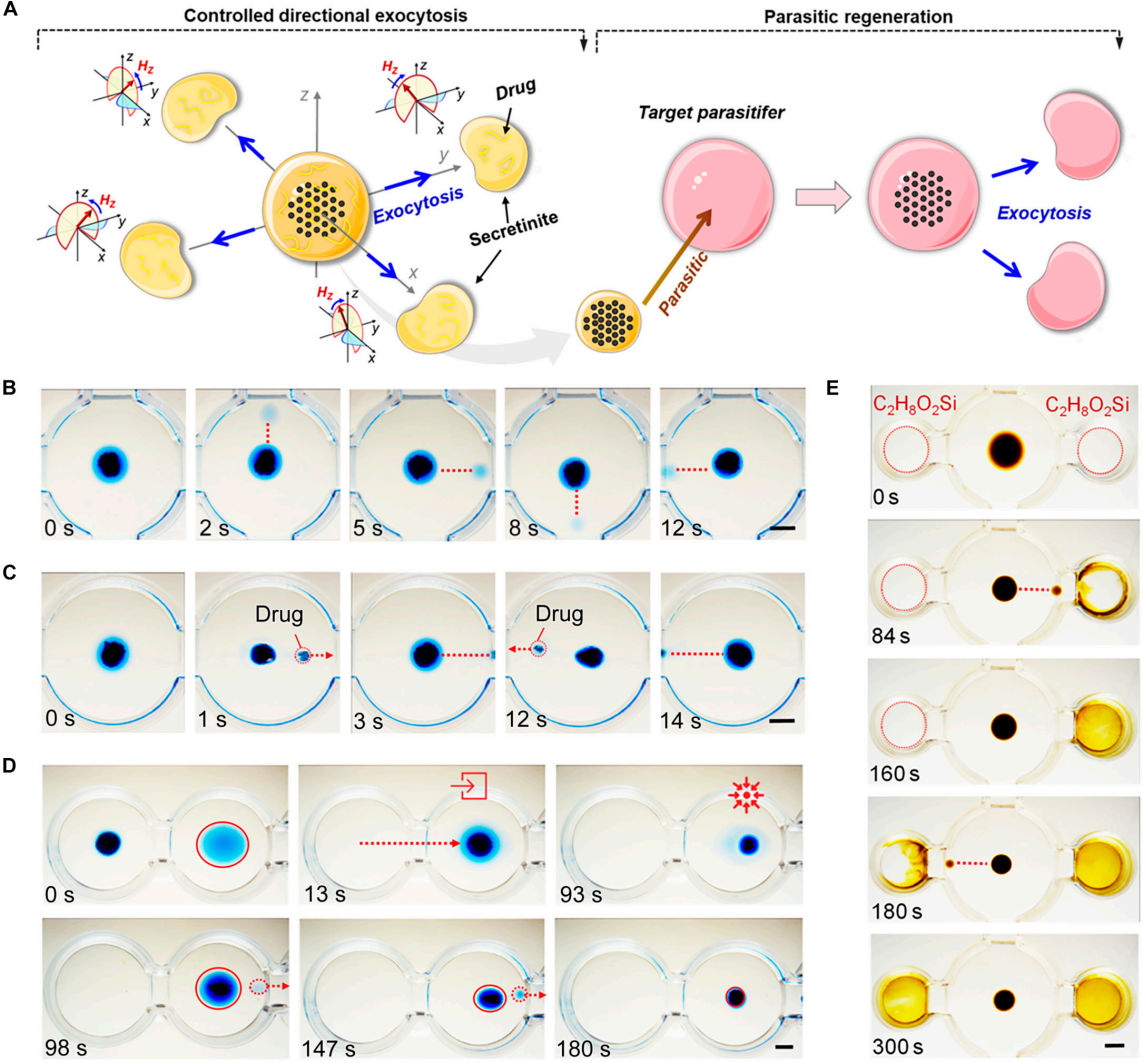
The application of intelligently regulated exocytosis behavior of cell-mimetic microrobots. (A) Flexible and controllable exocytosis of cell-mimetic microrobot. (B) Demonstration of secreting sub-droplets in any direction. Scale bars, 6 mm. (C) Repeated releasing of drugs outside a cell-mimetic droplet microrobot through exocytosis. Scale bar, 6 mm. (D) Manipulation of the microrobot to fragmentate a polluted oil droplet. The red circle indicates the shrinking contour line of the contaminated oil droplet. Scale bar, 4 mm. (E) Sequential dyeing of target reagent through exocytosis. The red dashed circle indicates the position of the silicone oil droplet. Scale bar, 6 mm.

The analysis above reveals that the magnetic field within the vertical plane is the primary factor in triggering and controlling the exocytosis behavior of cell-mimetic microrobot. Therefore, the exocytosis direction of the microrobots can be flexibly modulated by controlling the direction of the magnetic field in the vertical plane. This exocytosis performance of the microrobots can be continued by invading the parasitifer (Fig. 5A). The adjustability of the magnetic field enables the microrobot to emit sub-droplets one after another in programming directions (Fig. 5B and Movie S4). Interestingly, loaded with nonmagnetic substances, the exocytosis endows the microrobots with the ability to release cargoes in a targeted and noncontact manner. As demonstrated in Fig. 5C, the cell-mimetic microrobot, like a trebuchet, sequentially throws loaded drugs toward the target positions on both sides. We also explore the potential of using microrobots to treat floating oil pollution on the water surface presented in Fig. 5D and Movie S5. Guided by a gradient magnetic field, the microrobot first moves and invades the target contaminated oil (*t* = 13 s). Then, magnetic particles of the microrobot are further aggregated into compact disk-like swarm driven by an OSMF. Fragmentated into tiny biodegradable droplets through robotic exocytosis, the contaminated oil eventually disappears piece by piece from the water surface (*t* = 98 to 180 s). Figure 5E shows the dyeing process of target reagent through robotic secretion. The cell-mimetic microrobot loaded with staining agent keeps shooting droplets into the right vessel like a machine gun until the silicone oil inside is completely yellowed (*t* = 84 s). Then, it is the turn of the silicone oil in the left vessel to be shot and dyed yellow, which demonstrates the potential application of the microrobots in microchemical reactions and lab-on-a-chip devices.

### Controlled manipulation in gallbladder

**Fig. 6.**
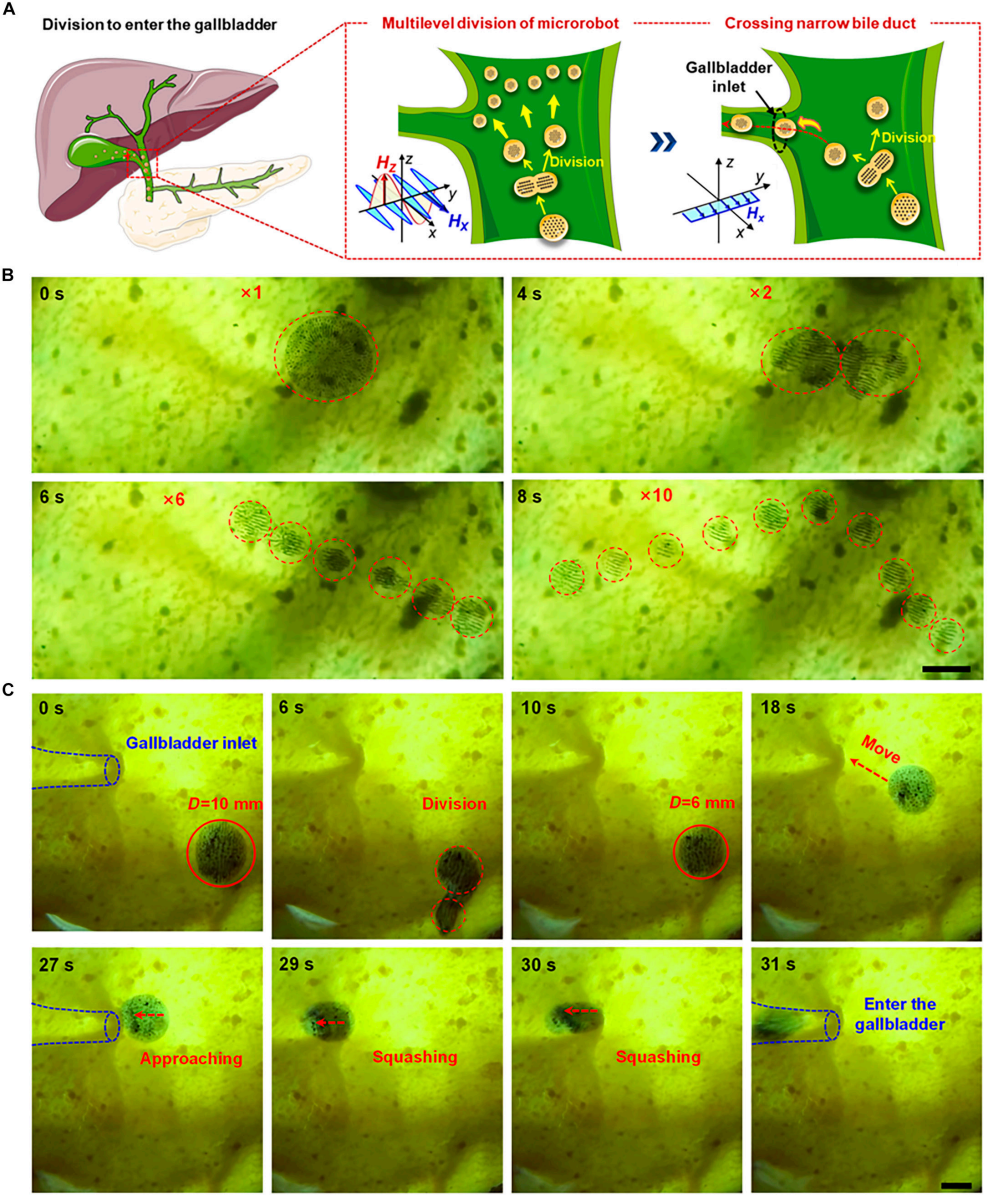
Division performance of cell-mimetic microrobots in gallbladder. (A) Schematic division behavior and crossing narrow channel modulated by OBMF. (B) One single cell-mimetic microrobot splits into 10 daughter cell-mimetic microrobots excited by OBMF. Scale bar, 4 mm. (C) The microrobot crosses the narrow channel of the gallbladder by splitting into smaller microrobots. Scale bar, 4 mm.

To better meet the practical application, the cell-mimetic microrobots are introduced into the bile duct through a clinical gastrointestinal endoscopy system and can be driven by an OBMF to guide the microrobots into the pig’s gallbladder in vitro. In this process, the cell-mimetic microrobots are regulated by the OBMF to split into small-sized cell-mimetic microrobots to pass through the narrow bile duct branch channel and enter the gallbladder (Fig. 6A). Figure 6B illustrates the process of cell-mimetic microrobot’s division, where a larger cell-mimetic microrobot (~10 μm) splits into 10 smaller microrobots (~2.5 μm) within 8 s under the modulation of orthogonal OSMFs (***A***_x_ = 5 mT, ***f***_x_ = 100 Hz, ***A***_z_ = 15 mT, ***f***_z_ = 10 Hz). As described in Movie S6, this division process simulates the behavior of cell division and is achieved by multiple divisions in steps.

The cell-mimetic microrobots’ controllable division performance also provides a new way for the microrobots to cross special tissue barriers in the body. A classic application scenario, as shown in Fig. 6C, demonstrates how the cell-mimetic microrobots can be magnetically controlled to divide, enabling it to pass through narrow channels that would otherwise be impassable in its original size. Initially, a cell-mimetic microrobot with a diameter of ~10 mm could not pass through the bile duct channel that was narrower than 3 mm. However, after applying OBMF, it splits into smaller microrobots with a size of ~6 mm. Subsequently, under the guidance of a gradient magnetic field, the smaller microrobots successfully pass through the narrow channel (Movie S7). Notably, the cell-mimetic microrobot undergoes a certain degree of deformation during passage, which is also a contributing factor to its ability to traverse the channel.

**Fig. 7.**
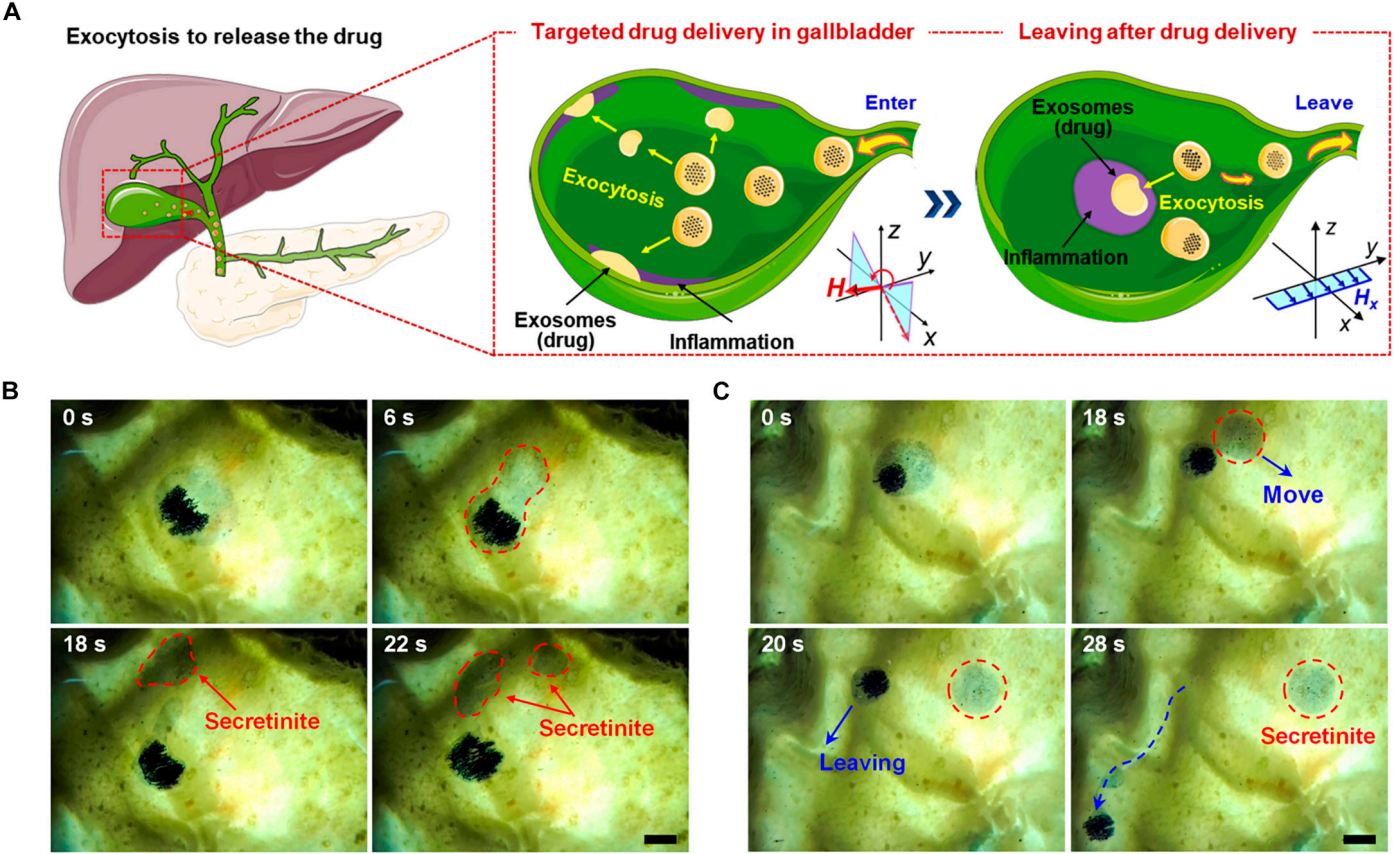
Exocytosis performance of cell-mimetic microrobots in gallbladder. (A) Schematic exocytosis behavior and drug delivery modulated by OSMF. (B) Continuous exocytosis behavior of cell-mimetic microrobot. Scale bar, 4 mm. (C) The microrobot leaves the gallbladder after releasing the drug through exocytosis. Scale bar, 4 mm.

After entering the gallbladder, the cell-mimetic microrobots can perform targeted drug release to the surrounding gallbladder wall through directional exocytosis. Additionally, following exocytosis, the cell-mimetic microrobots can be guided out of the gallbladder by a gradient magnetic field to prevent potential harm caused by residual magnetic particles (Fig. 7A). Figure 7B shows the cell-mimetic microrobot performing 2 consecutive exocytosis to release drug-loaded secretinite onto the inner wall of the gallbladder (Movie S8). Consistent with the exocytosis mechanism described in Fig. 4, the magnetic particle clusters inside the cell-mimetic microrobot aggregate to one side under the applied OSMF (***A***_x_ = 4 mT, ***f***_x_ = 1.0 Hz, ***A***_z_ = 12 mT, ***f***_z_ = 1.0 Hz). Subsequently, driven by the oscillating OSMF, the interaction between the microrobot and surface waves intensifies progressively. Once a critical threshold is reached, part of the “cytoplasm” detaches from the main body and moves toward the inner wall of the gallbladder. Figure 7C further illustrates the process of the cell-mimetic microrobot leaving the gallbladder after completing exocytosis and releasing the drug within the gallbladder (Movie S9). This ensures the safe application of the cell-mimetic microrobots by enabling it to leave the body after the drug delivery in the gallbladder is completed.

**Fig. 8.**
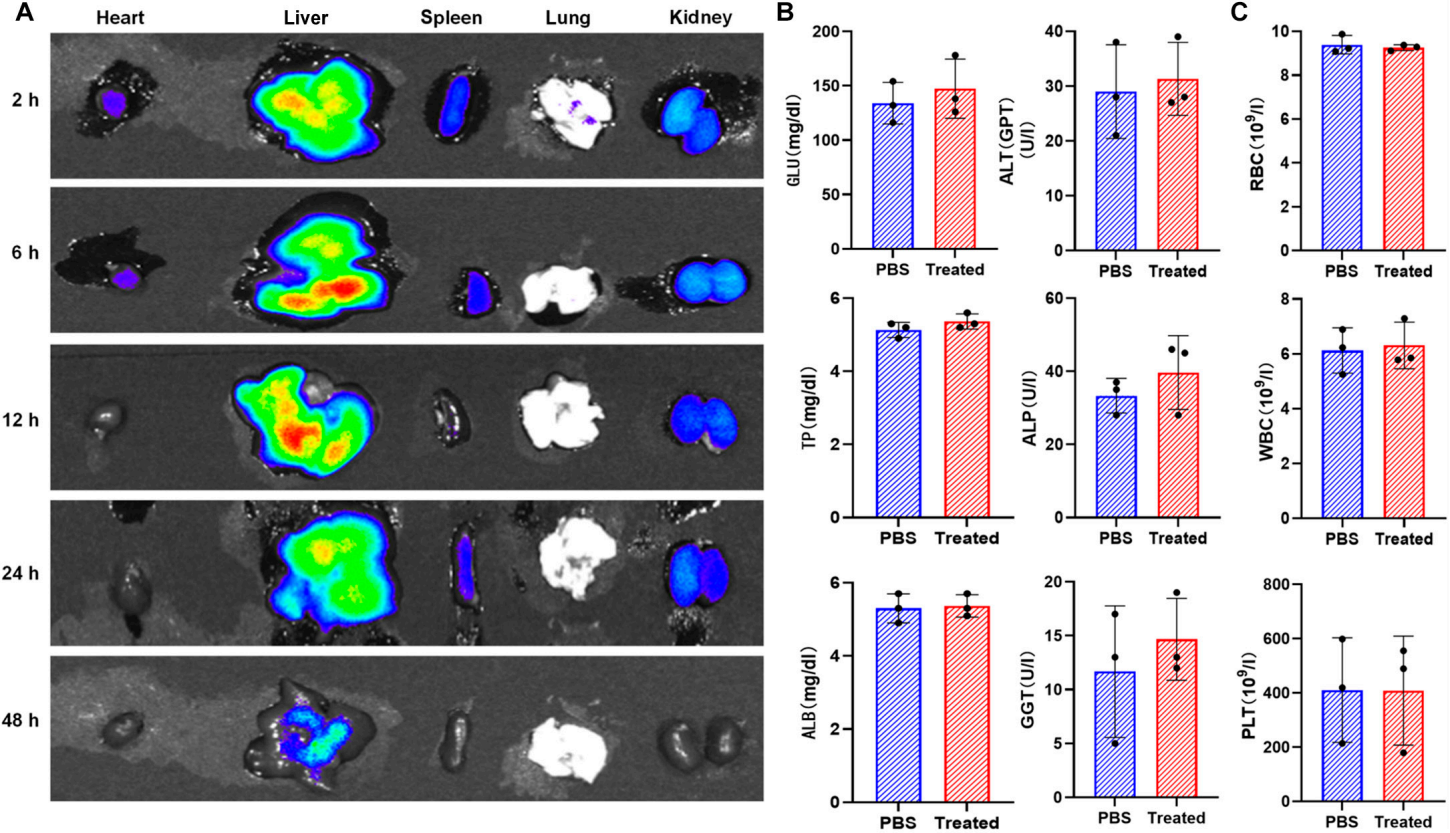
Biosafety analysis of cell-mimetic microrobots. (A) Fluorescence images showing the distribution of DIR within the heart, liver, spleen, lung, and kidney of mice treated by cell-mimetic microrobots for 2, 4, 12, 24, and 48 h. (B) Biochemical index and (C) blood chemistry panel taken from mice treated by cell-mimetic microrobots and PBS.

In order to evaluate the biosafety analysis of cell-mimetic microrobots, we employed in vivo fluorescence imaging to monitor the influence of the cell-mimetic microrobots on the body’s metabolic processes. As shown in Fig. 8A, at 2 h post-administration, the fluorescence signal was strong and clearly distributed throughout the observed regions. As time progressed, both the intensity and distribution of the fluorescence signal began to change noticeably at 6 and 12 h. By 24 h, the fluorescence in certain areas had further diminished, and at 48 h, the fluorescence signal was significantly reduced. These observations indicate that the fluorescently labeled components underwent a distinct metabolic process within the body. Based on the substantial decrease in signal over time, it can be inferred that the majority of the material was metabolized within 48 h. The marked reduction in fluorescence provides intuitive visual evidence of the material’s metabolic clearance trend, suggesting that the substance exhibits a reasonable metabolic rate and is largely eliminated by 48 h. This result highlights the body’s strong ability to clear the cell-mimetic microrobot material, offering direct imaging-based support for its favorable biosafety profile and metabolic compatibility.

Further, we conducted hematological and biochemical analyses to assess the biosafety of the cell-mimetic microrobots following endoscopic administration into the gallbladder. As shown in Fig. 8B and C, the results indicate that most indicators exhibited no significant differences between the phosphate buffer solution (PBS)-treated group and the experimental group, suggesting that the microrobot administration did not cause notable hematological or biochemical disturbances. Regarding biochemical markers, indices reflecting nutritional and metabolic status, such as total protein (TP) and albumin (ALB), showed no significant fluctuations, suggesting only minimal impact on basic metabolic functions. Although some parameters exhibited slight variations, they remained within the normal physiological range and showed no sustained abnormal trends. For example, certain enzyme indicators [such as alanine aminotransferase (ALT) and alkaline phosphatase (ALP)] displayed minor fluctuations but did not exceed normal reference values, implying that liver and kidney functions were not significantly affected. In terms of hematological parameters, indicators such as red blood cells (RBCs) and white blood cells (WBCs) remained relatively stable between the 2 groups, indicating that the microrobots did not induce significant abnormalities in blood cell counts. Overall, these results provide preliminary evidence that the microrobots possess good biosafety and do not induce obvious systemic hematological or biochemical abnormalities.

## Discussion

Our results demonstrate that surface wave-assisted droplet microrobots have the potential to construct robotic systems with vitality that replicate diverse life activities of cellular organisms. Using OBMFs, the strategies presented here program magnetic particle swarms encapsulated in the cell-mimetic microrobots into multiple dynamic formations, classified as gas, chain, array, and disk-shaped collective patterns. Accordingly, the cell-mimetic microrobots floating at the air–water interface are activated by reversible swarm transitions between modes and reproduce the unique life activities of living cells, such as mitotic division and cell secretion. Moreover, the physical mechanisms governing the dynamics of living robotic systems have also been carefully investigated, revealing that the remarkable vitality of cell-mimetic microrobots is directly related to surface waves triggered by the magnetic response of particle collectives. The division and exocytosis performance of the cell-mimetic microrobots inside the gallbladder demonstrated their strong potential for navigating tissue barriers and achieving targeted drug delivery within biological systems.

We believe that this method is feasible in further in vivo experiments. There are no other ferromagnetic materials in biological system, so magnetic shielding will not occur, and there will be no marked attenuation of magnetic field strength. This ensures that there are no feasibility barriers for magnetically driven and navigated cell-mimetic microrobot. However, the research on cell-mimetic microrobot, and indeed most micro-robots, is still at the experimental stage. The obstacles that need to be overcome for practical clinical application should be recognized: (a) Imaging and tracking technologies within the biological system need further improvement to ensure precise navigation and operation of the microrobots. (b) The strength and flexibility of the magnetic field need to be enhanced to enable more durable and versatile driving and control mechanisms. (c) The complexity of the microrobots should also be increased, requiring designs and functionalities that are as universal as possible. To date, several types of imaging techniques (such as photoacoustic imaging [54] and magnetic particle imaging [55]) have been developed and tested to visualize microrobots in vivo. Our future research will focus on the development of more flexible magnetic field systems, such as mechanically adjustable magnetic field generator arrays mounted on robotic arms, combined with advanced imaging technologies for in vivo experiments in robotic applications. Additionally, the cell-mimetic microrobot in this study is designed to the millimeter scale, which can better demonstrate the principles of deformation, division, and exocytosis while meeting the navigation drive and operation in the gallbladder environment. However, the size of cell-mimetic microrobots is not strictly limited to the millimeter scale. For applications in more confined environments, micrometer-scale microrobots could be fabricated through magnetic field control. The feasibility of this approach is preliminarily validated by the reproducible multistage division shown in Figs. 3F and 6B. We will continue to advance research on the flexibility and adaptability of cell-mimetic microrobots to meet the diverse demands of various disease treatments.

## Materials and Methods

### Materials and experimental details

The preparation of magnetic cell-mimetic microrobots is illustrated in Fig. 1A. Firstly, a drop of n-dodecane is injected onto the surface of deionized water in a glass container. Based on the experimental methods, the minimum volume of oil droplets involved was 2.0 μl. Then, nickel microparticles (average diameter of 100 μm, Ni, Nangong Fufan Metal Materials Co. Ltd., China) are added into the floating oil droplet to generate a droplet microrobot. The morphology analysis and magnetic hysteresis properties of magnetic microparticles are shown in Figs. S4 and S5. The mass concentration of droplet microrobots in most experiments is 0.8 g/ml, except for the exocytosis demonstrations shown in Fig. 4, which are carried out in high concentrations up to 1.8 g/ml. After that, the magnetic droplet was transferred to the working space of the electromagnetic setup for further magnetic actuation processes. Before the formal experiments, magnetic particles concentrated in the oil droplets must be sputtered and spread flat on the oil–water interface using the vibrating magnetic field illustrated in Fig. 2A. Notably, experimental result in Figs. S6 and S7 presented that the cell-mimetic microrobots exhibit very similar magnetic field response parameters and behavioral patterns for division and exocytosis in both bile and deionized water. For ease of observation, deionized water was used in the subsequent experiments.

### Electromagnetic actuation platform

All the biomimetic operations of cell-mimetic microrobots were actuated by orthogonal vibration magnetic field excited in the triaxial Helmholtz coil, which is controlled by a group of oscillating signal inputs. Figure S8 shows the magnetic field drive setup and the drive process. The control signals were triggered by PC via a LabVIEW program and then amplified by current amplifiers to obtain sufficient field strength up to 40 mT. In magnetic actuation experiments, the cell-mimetic microrobots were surrounded by the Helmholtz coils mounted on an optical microscope. By controlling the input current of Helmholtz coils, the external magnetic field can be produced and programmed in the workspace to construct the cell-inspired robotic system in desired modes.

### Division and exocytosis in gallbladder

The experiment was conducted using a pig gallbladder (Experimental Animal Center of the First Affiliated Hospital of Harbin Medical University), and the process was recorded using a charge-coupled device (CCD) camera. The gallbladder, obtained from the pig, was carefully washed with physiological saline and then immersed in a 4% PFA (paraformaldehyde) preservation solution (Beijing Biotopped Life Sciences Inc., China). The gallbladder was then dissected, and droplet microrobots were prepared inside using light fluorinated oil (GALDEN DET, Italy) and F16CuPc (Shanghai Aladdin Biochemical Technology Inc., China), which facilitated the coloration of the cell-mimetic microrobots. To ensure optimal performance in the gallbladder environment, 2 to 3 ml of deionized water were added to the inner wall of the gallbladder to promote wetting. For the division and exocytosis experiments, the concentration of the droplet microrobots was adjusted to 0.8 and 1.8 g/ml, as shown in Figs. 6 and 7. As mentioned earlier, the droplet microrobots successfully performed the division and exocytosis processes inside the gallbladder under the influence of the magnetic field.

### Organ metabolism analysis

Healthy mice were selected and adaptively raised for several days to ensure that their physiological state was stable for subsequent experiments. DIR was then mixed with magnetic fluid and accurately introduced into the gallbladder area of the mouse through an endoscope. Imaging was performed using a small animal in vivo imaging system 2, 6, 12, 24, and 48 h after administration. The mice were anesthetized before the operation to keep them quiet, and then the mouse viscera were dissected and fixed on the imaging platform and adjusted to a uniform position. According to the characteristics of the fluorescent marker, the appropriate excitation wavelength and emission wavelength were set to ensure that the imaging conditions were consistent each time. After shooting, the fluorescence images at each time point were recorded in detail, and the intensity and spatial distribution changes of the fluorescence signal were analyzed to evaluate the metabolic process and characteristics of the substance in the body.

### Biochemical and blood index analysis

Healthy mice were selected to establish a gallbladder-related research model to ensure that they adapted to the experimental environment and entered the experimental stage after their physiological state was stable. Subsequently, the cell-mimetic microrobots were accurately introduced into the gallbladder through an endoscope, and a PBS-treated group was set as a control to simulate the drug administration operation but only give an equal amount of PBS. At a specific time point after administration, blood samples from animals were collected and placed in blood collection tubes containing anticoagulants (for blood routine tests) and without anticoagulants (for biochemical analysis), and serum/plasma and blood cells were centrifuged. Finally, a fully automatic blood cell analyzer was used to detect RBC, WBC, PLT (platelets), and other indicators. A fully automatic biochemical analyzer was used to detect biochemical indicators such as TP, ALB, ALT, and ALP. The results of each test are shown in Fig. 8 and Fig. S9. The abbreviations and reasonable ranges of various detection indices are shown in Table S1.

### Simulation analysis

The simulations were performed within the framework of COMSOL Multiphysics 6.1. Computational fluid dynamics (CFD) simulations were performed in a 2-dimensional plane by solving the Navier–Stokes equations for microrobots placed in a rectangular microfluidic channel filled with deionized water as media. The magnetic particles were regarded as an array of oscillating spheroids, inducing the fluidic velocity field. The oscillation frequency of the spheroids in the simulation matches the frequency of the applied magnetic field. The results were visualized using normalized arrows to indicate the flow direction, while the color intensity represented the flow rate.

## Data Availability

All data required to evaluate the conclusion of the paper is presented in this paper and Supplementary Materials. Additional data related to this article can be reasonably obtained from the authors.
