# Supplementary material for: Magnetic Cell-Mimetic Droplet Microrobots with Division and Exocytosis Capabilities
Source: Research (Wash D C). 2025 Jun 3;8:0730. doi: 10.34133/research.0730 (PMC12130622; doi:10.34133/research.0730)
Supplement: Supplementary 1 — Figs. S1 to S9 Table S1 Movies S1 to S9 [file research.0730.f1.zip › Supplementary_Materials.docx]

Supplementary Materials for

**Magnetic Cell-Mimetic Droplet Microrobots with Division and Exocytosis Capabilities**

Shimin Yu^1,†^, Weiwei Zhang^2,†,^*, Yongzhi Feng^3,†^, Xiang Zhang^4^, Shengjun Shi^3^, Haocheng Wang^3,^*, Tianlong Li^3,^*

*Corresponding author. Email: vivid@zzu.edu.cn; wanghaocheng@hit.edu.cn; tianlongli@hit.edu.cn

**This PDF file includes:**

Fig. S1. The variation of the average distance between chains ***d***_chain_ with the magnitude of (A) ***A***_z_ and (B) ***A***_x_.

Fig. S2. Schematic diagram of (A) orthogonal butterfly magnetic field (OBMF) and (B) orthogonal oscillating magnetic field (OSMF) used to trigger the cell-mimetic droplet microrobots to generate division and exocytosis behaviors, respectively.

Fig. S3. Reassembly of divisive cell-mimetic microrobots by switching magnetic field direction in the horizontal plane.

Fig. S4. The morphology analysis magnetic particles used in cell-mimetic microrobots.

Fig. S5. Magnetic hysteresis curve of magnetic particles with average diameter of 100 μm.

Fig. S6. The division of cell-mimetic microrobot in culture medium containing bile.

Fig. S7. The exocytosis of cell-mimetic microrobot in culture medium containing bile..

Fig. S8. Magnetic field drive setup.

Fig. S9. Biochemical index taken from mices treated by cell-mimetic microrobots and PBS.

Table S1. The abbreviations and reasonable ranges of various detection indexs.

**Other Supplementary Materials for this manuscript include the following:**

Movie S1. The division process of cell-mimetic microrobot triggered by internal particle cluster mode changes.

Movie S2. Demonstration of repeated robotic division.

Movie S3. The exocytosis process of a cell-mimetic microrobot energized by butterfly oscillation fields.

Movie S4. Demonstration of secreting sub-droplets in any direction.

Movie S5. Manipulation of the robot to fragmentate a polluted oil droplet.

Movie S6. One single cell-mimetic microrobot splits into 10 daughter cell-mimetic microrobots.

Movie S7. The robot crosses the narrow channel of the gallbladder by splitting into smaller robots.

Movie S8. Continuous exocytosis behavior of cell-mimetic microrobot in gallbladder.

Movie S9. The microrobot leaves the gallbladder after releasing the drug through exocytosis.


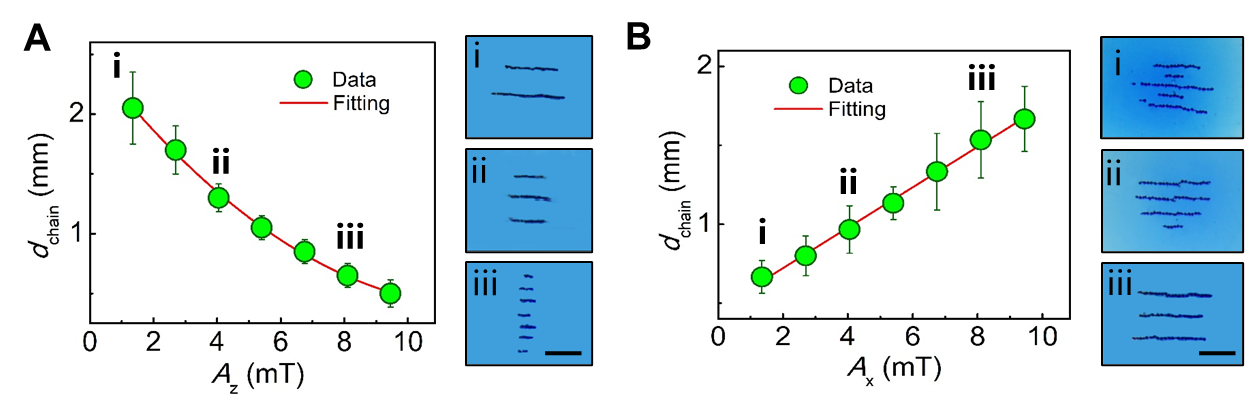


**Fig. S1.** The variation of the average distance between chains ***d***_chain_ with the magnitude of (A) ***A***_z_ and (B) ***A***_x_. (Reviewer 2, Comment 2)


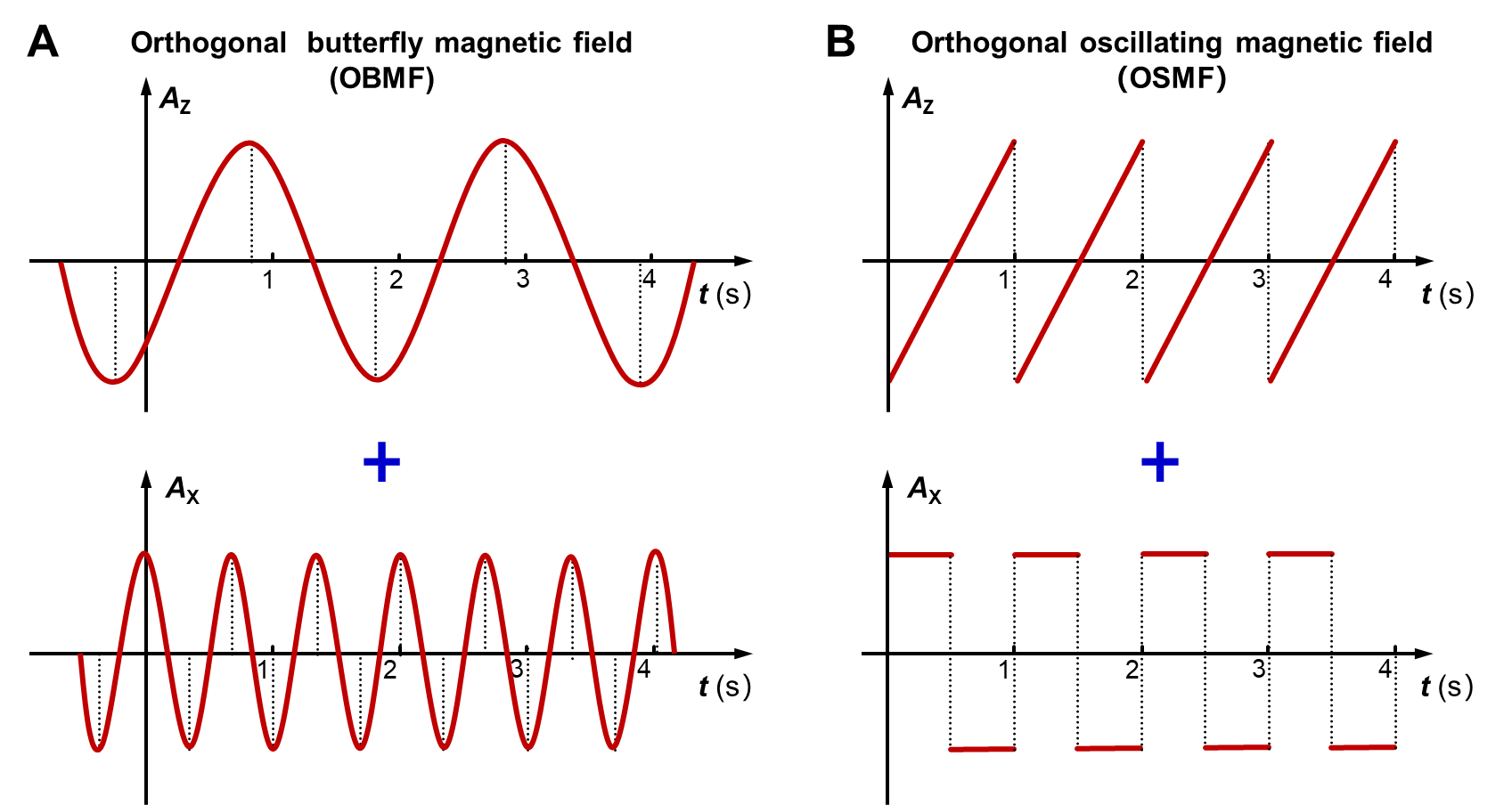


**Fig. S2.** Schematic diagram of (A) orthogonal butterfly magnetic field (OBMF) and (B) orthogonal oscillating magnetic field (OSMF) used to trigger the cell-mimetic droplet microrobots to generate division and exocytosis behaviors, respectively. (Reviewer 1, Comment 2) (Reviewer 2, Comment 1) (Reviewer 3, Comment 1)


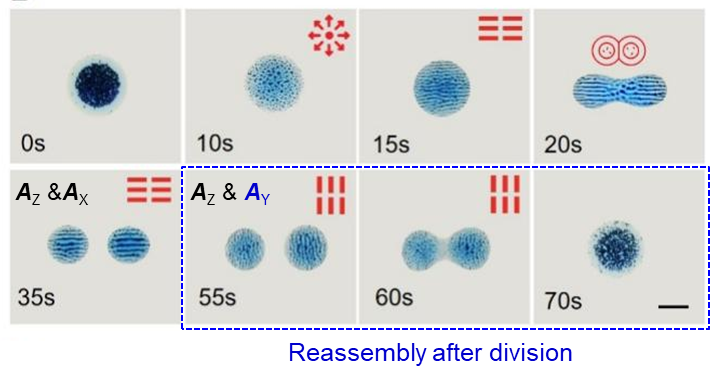


**Fig. S3.** Reassembly of divisive cell-mimetic microrobots by switching magnetic field direction in the horizontal plane. (Reviewer 1, Comment 4)


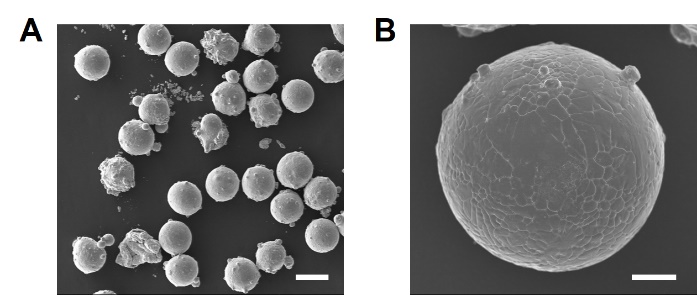


**Fig. S4.** The morphology analysis magnetic particles used in cell-mimetic microrobots. A) The SEM image of a cluster of magnetic particles. The scale bar is 100 μm. B) The SEM image of one particle. The microparticles have an average diameter of 100 μm. The scale bar is 20 μm. (Reviewer 3, Comment 2)


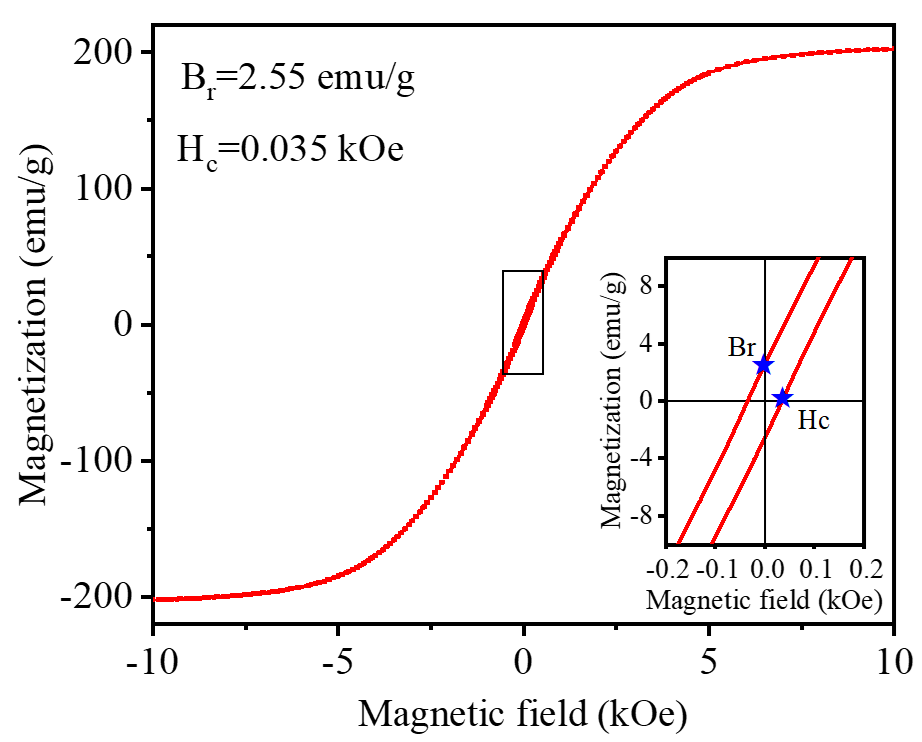


**Fig. S5.** Magnetic hysteresis curve of magnetic particles with average diameter of 100 μm. (Reviewer 3, Comment 2)


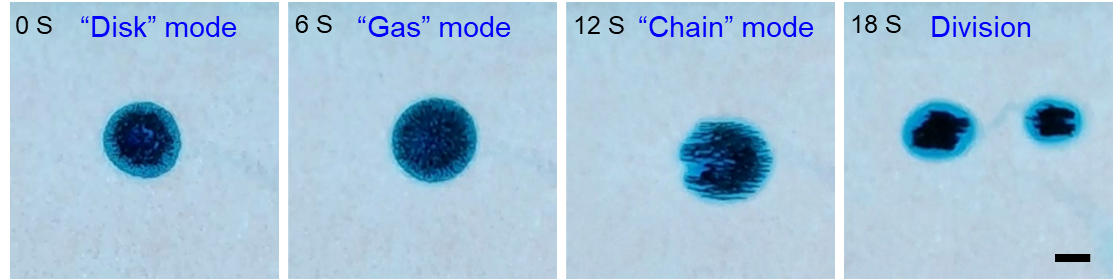


**Fig. S6.** The division of cell-mimetic microrobot in culture medium containing bile. The scale bar is 4mm. (Reviewer 2, Comment 5)


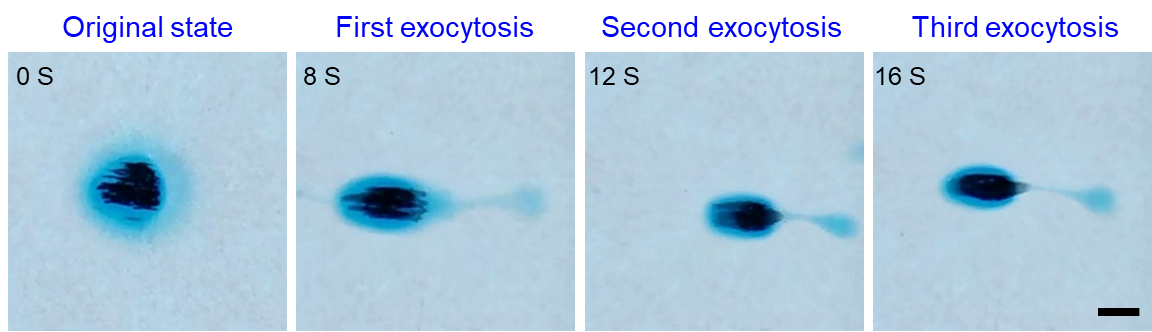


**Fig. S7.** The exocytosis of cell-mimetic microrobot in culture medium containing bile. The scale bar is 4mm. (Reviewer 2, Comment 5)


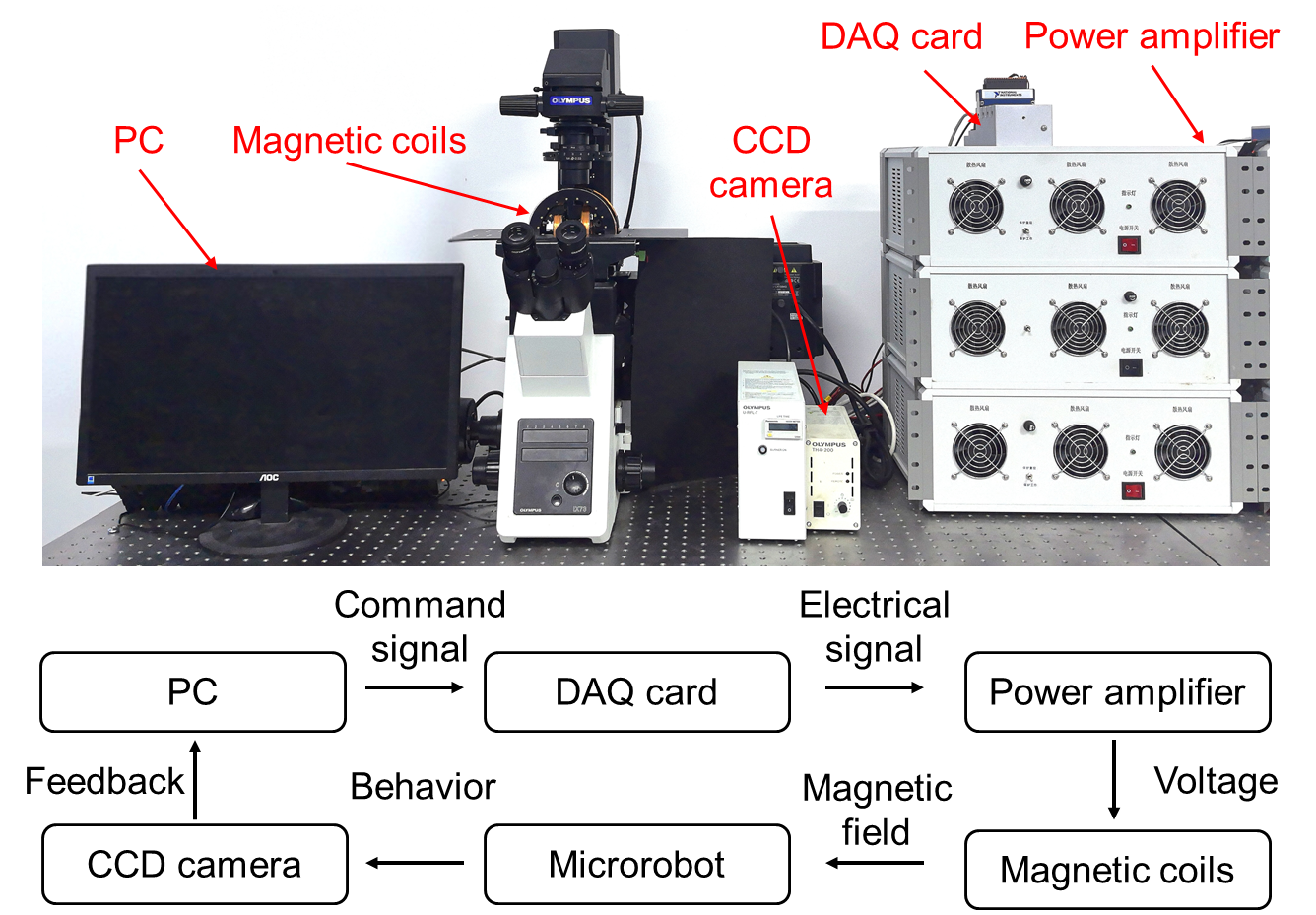


**Fig. S8.** Magnetic field drive setup. Motion commands were provided by the PC terminal and executed by the magnetic coil. The data on the motion behaviors of cell-mimetic microrobots were coupled back to the PC terminal. (Reviewer 3, Comment 4)


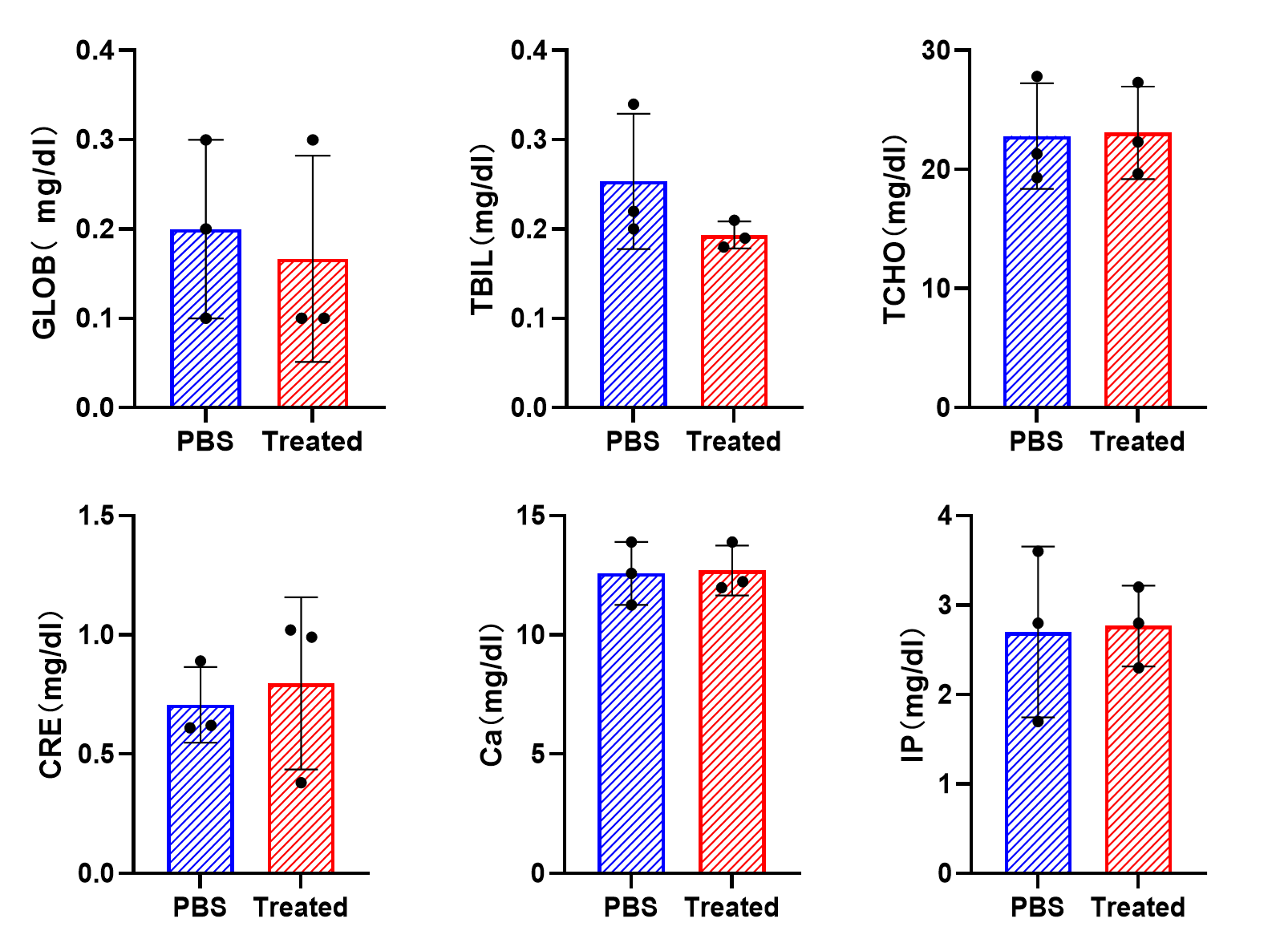


**Fig. S9.** Biochemical index taken from mices treated by cell-mimetic microrobots and PBS. (Reviewer 2, Comment 4)

**Table S1.** The abbreviations and reasonable ranges of various detection indexs. (Reviewer 2, Comment 4)

| Index | Abbreviation | Reasonable ranges |
| --- | --- | --- |
| Glucose | GLU | 115~214 mg/dl |
| Total protein | TP | 4.9~6.9 g/dl |
| Albumin | ALB | 4.6~6.3 g/dl |
| Globulin | GLOB | 1.8-3.5 mg/dl |
| Total bilirubin | TBIL | 0.1~0.4 mg/dl |
| Total cholesterol | TCHO | 11~74 mg/dl |
| Alkaline phosphatase | ALP | 21~75 U/I |
| Alanine aminotransferase | ALT (GPT) | 12~72 U/I |
| γ-glutamyl transpeptides | GGT | 5~18 U/I |
| Creatinine | CRE | 0.60~1.40 mg/dl |
| Calcium | Ca | 12.5~14.5 mg/dl |
| Phosphorus | IP | 1.6~4.1 mg/dl |
| Red blood cell | RBC | \ |
| White blood cells | WBC | \ |
| Platelets | PLT | \ |
